# The New Reliable pH Sensor Based on Hydrous Iridium Dioxide and Its Composites

**DOI:** 10.3390/ma16010192

**Published:** 2022-12-25

**Authors:** Nikola Lenar, Robert Piech, Beata Paczosa-Bator

**Affiliations:** Faculty of Materials Science and Ceramics, AGH University of Science and Technology, Mickiewicza 30, PL-30059 Krakow, Poland

**Keywords:** pH sensing, solid-contact electrodes, iridium dioxide, composite materials, carbon nanotubes, poly(3-octylthiophene-2,5-diyl)

## Abstract

The new reliable sensor for pH determination was designed with the use of hydrous iridium dioxide and its composites. Three different hIrO_2_-based materials were prepared and applied as solid-contact layers in pH-selective electrodes with polymeric membrane. The material choice included standalone hydrous iridium oxide; composite material of hydrous iridium oxide, carbon nanotubes, and triple composite material composed of hydrous iridium oxide; carbon nanotubes; and poly(3-octylthiophene-2,5-diyl). The paper depicts that the addition of functional material to standalone metal oxide is beneficial for the performance of solid-state ion-selective electrodes and presents the universal approach to designing this type of sensors. Each component contributed differently to the sensors’ performance—the addition of carbon nanotubes increased the electrical capacitance of sensor (up to 400 µF) while the addition of conducting polymer allowed it to increase the contact angle of material changing its wetting properties and enhancing the stability of potentiometric response. Hydrous iridium oxide contacted electrodes exhibit linear response in wide linear range of pH (2–11) and stable potentiometric response (the lowest potential drift of 0.036 mV/h is attributed to the electrode with triple composite material).

## 1. Introduction

The quantity pH is intended to be a measurement of the activity of hydrogen ions in a solution [1]. According to IUPAC recommendations [2], potentiometry with the use of hydrogen-sensitive electrodes is the only accurate method of pH measurement. Conventionally, pH measurements are performed with the use of a glass electrode [2,3,4,5,6].

The glass electrode was the first electrode acknowledged as an ion-selective electrode designed by Klemensiewicz and Haber [7], who recognized the practical application coming from the research on glass membranes performed by Cremer [8]. Since invented in the 1900s, the glass electrode has been widely used for pH detection and was unlikely to be replaced because of its great analytical parameters [5,6,9]. The glass electrode belongs to the oldest group of potentiometric sensors named conventional electrodes, characterized with the presence of the internal solution. This type of an electrode requires vertical position and stable temperature and pressure to avoid the phase change of an inner solution [10]. Moreover, the presence of glass bubble, acting as a pH-selective membrane, makes the glass electrode brittle and vulnerable to mechanical damage [5]. Because of those disadvantages, coming from the electrode’s construction rather than the poor analytical performance, the glass electrode is likely to find its substitute in all-solid-state, ion-selective electrodes.

The first electrode representing this new group was designed by Cattrall and Freiser in 1971 [11] and was called the coated wire electrode as the ion-selective membrane was coated around the metallic wire and later also on the other types of electrodes, such as disc electrodes. This type of an electrode allowed it to overcome all disadvantages related to the presence of an inner solution and the problem of brittleness; however, the analytical performance was unfortunately deteriorated. It later turned out that the indirect contact of an electrode and ion-selective membrane, which is electronic and ionic conductor, causes the blockage of the charge transfer resulting in high resistance and deterioration of potential stability. The stability is reportedly limited by the small (double-layer) capacitance formed at the interface between the electronic conductor and the ion-selective membrane [12]. 

The potential stability of the all solid state electrode can be improved by applying an intermediate layer between the electronic and ionic conductor with suitable ion-to-electron transduction properties [13]. This group of electrodes was named solid-contact electrodes as the solid-contact layer is placed in-between the electronic conductor and ion-selective membrane. This group of electrodes developed rapidly over the last 30 years, and nowadays, solid-contact electrodes are characterized by analytical parameters nearly as excellent as those of the glass electrode [3,14].

Various materials have been applied over the years as solid-contact layers in pH-sensitive ion-selective electrodes including conducting polymers poly(3,4-ethylenedioxythiophene)/poly(styrenesulfonate) PEDOT/PSS [15] and derivative of poly(3,4-ethylenedioxythiophene) PEDOT-C_14_ [16], carbon nanomaterials such as multiwalled carbon nanotubes MWCNTs [17], and hydrous metal oxides—ruthenium dioxide hRuO_2_ [14]_._

This study focuses on introducing the new materials based on hydrous iridium dioxide into pH-selective solid-contact electrodes with polymeric membrane. Three different materials were introduced in the scope of this paper including standalone hydrous iridium dioxide (hIrO_2_), double composite of iridium dioxide with carbon nanotubes (hIrO_2_-NTs) and triple composite of iridium dioxide, carbon nanotubes and poly(3-octylthiophene-2,5-diyl) (hIrO_2_-NTs-POT). The last material is a unique combination of three significantly different materials: metal oxide, carbon nanomaterial, and conducting polymer into one composite material applied for the first time as solid-contact layer in pH-selective sensors.

## 2. Materials and Methods

### 2.1. Materials

Aqueous solutions were prepared by dissolving salts and acids in distilled and deionized water. Standard solutions of fixed pH value were prepared by dissolving citric acid (POCH, Gliwice, Poland) and boric acid (POCH). The buffer solution of 1 mM citric acid and 1 mM boric acid was titrated with 1M sodium hydroxide and 1M hydrochloric acid. NaOH (POCH) and HCl (POCH) were added to meet the desired pH value that is 4-12 and 2-3, respectively. All chemicals used for solution preparation were of analytical grade and were used as received without any further purification.

Designed pH sensors are ion-selective electrodes characterized by a sandwich structure with ion-selective membrane placed on the solid-contact layer. The solid-contact layer consisted of hydrous iridium dioxide (hIrO_2_) (Alfa Aesar, Haverhill, MA, USA), multiwalled carbon nanotubes (NTs) (Nanostructured & Amorphous Materials, Inc., Houston, TX, USA), and Poly(3- octylthiophene-2,5-diyl) (POT) (Sigma Aldrich, St. Louis, MO, USA). The ion-selective membrane consisted of hydrogen ionophore V (Calix[4]-aza-crown), sodium tetrakis(4-fluorophenyl)borate dihydrate, 2-Nitrophenyl octyl ether (NPOE), and poly(vinyl chloride) (PVC) of high molecular weight were purchased from Sigma-Aldrich. Dimethylformamide (DMF) and Tetrahydrofuran (THF) used as solvents were also purchased from Sigma-Aldrich.

### 2.2. Sensor’s Preparation

Designed pH sensors are solid-contact ion selective electrodes with pH-selective membrane responsible for selective recognition of hydrogen ions and solid-contact layer between the membrane and electrode’s surface.

The preparation procedure starts with preparation of glassy carbon disc (GCD) electrodes’ surface by polishing the disc using aluminum oxide paste of descending grain size and rinsing it with deionized water and methanol. 

For the purpose of this work 12 items of pH-selective electrodes were prepared: 3 items with hydrous iridium dioxide layer (hIrO_2_); 3 items with double composite layer of hydrous iridium dioxide and carbon nanotubes (hIrO_2_ – NTs); 3 items with triple composite layer of hydrous iridium dioxide, carbon nanotubes, and Poly(3- octylthiophene-2,5-diyl) (hIrO_2_ – NTs – POT); and 3 items without solid-contact layer used as a control group.

Material for hydrous iridium dioxide layer was prepared by ultrasonically dispersing (for 30 min) 7 mg of hIrO_2_ in 1 mL of DMF. Double composite material was prepared by ultrasonically dispersing (for 30 min) 5 mg of hIrO_2_ and 10 mg of carbon nanotubes (NTs) in 1 mL of DMF. The triple composite material was prepared by ultrasonically dispersing (for 15 min) 5 mg of hIrO_2_, 5 mg of NTs, and 10 mg of POT in 1mL of THF. The solution was then centrifuged for 15 min (10 000 RPM), and then, the sediment of solid particles was separated. After centrifugation, the portion of the POT dissolved in THF was removed, and the residue after centrifugation (hIrO_2_, NTs and undissolved POT) was dispersed again ultrasonically (15 min) in a new amount of THF (1 mL).

Electrodes representing each group were casted with a pH-selective membrane. The membrane was prepared by dispersing 252 mg of the membrane components in 2 mL of THF. The composition of pH-selective membrane was as follows: 0.90% (*w*/*w*) hydrogen ionophore V, 66% (*w*/*w*) o-NPOE, 32.85% (*w*/*w*) PVC, and 0.25% (*w*/*w*) sodium tetrakis(4-fluorophenyl)borate dihydrate.

All electrodes were prepared with the use of drop-casting method following the procedure for each group listed below: 

1—GC/hIrO_2_/H^+^-ISM sensors were prepared by casting 15 µL of hIrO_2_ solution onto disc electrode surface, drying the solution in 80 degrees until the solvent (DMF) evaporation and then casting the obtained solid-contact hIrO_2_ layer with 60 µL of membrane solution.

2—GC/hIrO_2_+NTs/H^+^-ISM sensors were prepared by casting 15 µL of hIrO_2_-NTs solution onto disc electrode surface, drying the solution in 80 degrees until the solvent (DMF) evaporation and then casting the obtained solid-contact hIrO_2_-NTs layer with 60 µL of membrane solution.

3—GC/hIrO_2_+NTs+POT/H^+^-ISM sensors were prepared by casting 15 µL of hIrO_2_-NTs-POT solution onto disc electrode surface, drying the solution in room temperature until the solvent (THF) evaporation, and then casting the obtained solid-contact hIrO_2_-NTs-POT layer with 60 µL of membrane solution.

Drop-casting method is simple and fast, and no additional binder is required when using this technique as the membrane’s solid particles adhere to the electrode (or mediation layer, in case of solid-contact electrodes) after the solvent (THF) evaporation.

All prepared sensors were conditioned in the buffer solution of pH 3 prior to every measurement.

### 2.3. Conducted Measurements

Potentiometric measurements were performed using a 16- channel mV-meter (Lawson Labs, Inc., Malvern, PA, USA). The potentiometric response towards H^+^ ions was examined in the standard solutions buffered with 10 mM citric acid and 10 mM boric acid titrated with sodium hydroxide or hydrochloric acid. The pH values of buffer solutions were fixed on a value from 2 to 12. Potentiometric measurements were conducted versus the reference electrode-Ag/AgCl electrode with 3 M KCl solution (6.0733.100 Metrohm, Herisau, Switzerland) and in the presence of auxiliary electrode-platinum wire.

The chronopotentiometric measurements were carried out with the use of an Autolab General Purpose Electrochemical System (AUT302N.FRA2-AUTOLAB, Metrohm Autolab, Barendrecht, The Netherlands) with NOVA 2.1. software. Designed pH-selective sensors ion-selective electrodes were tested as working electrodes in three-electrode cell with a reference electrode Ag/AgCl electrode with 3 M KCl solution (6.0733.100 Metrohm, Herisau, Switzerland) and in the presence of auxiliary electrode–platinum wire. Chronopotentiometric tests were conducted in the standard buffer solution of pH 3. A constant current of +100 nA was applied to the working electrode for 60 s, followed by a −100 nA current for another 60 s according to the procedure proposed by Bobacka [12].

The examination of the wetting properties of the materials for solid-contact layers was performed with the use of the Theta Lite contact angle microscope with One Attension software by Biolin Scientific, Frölunda, Sweden. 

The microstructure of solid-contact materials was examined using Scanning Electron Microscope and Transmission Electron Microscope. SEM scans were collected using Scanning Electron Microscope-LEO 1530 (Carl Zeiss, Germany) and TEM scans using Transmission Electron Microscope—Tecnai 20 X-TWIN (FEI, Hillsboro, OR, USA).

## 3. Results

### 3.1. Materials’ Microstructure Characteristics 

**Figure 1:**
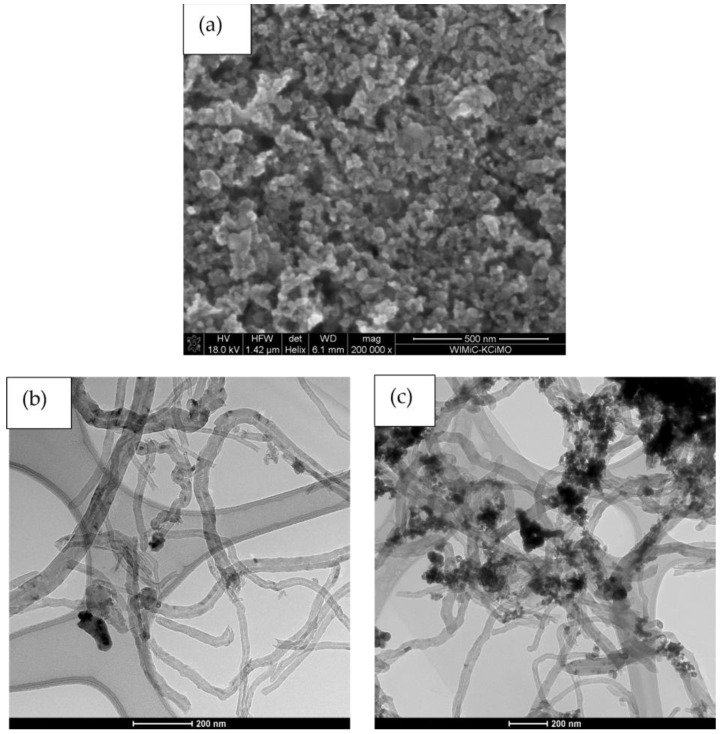
Microstructure of solid-contact materials: (**a**)—SEM scan of IrO_2_, (**b**)—TEM scan of IrO_2_-NT, (**c**)—TEM scan of IrO_2_-NT-POT.

The microstructures of materials for solid-contact layers were examinated using Scanning and Transmission Electron Microscope. SEM scan of iridium dioxide (Figure 1a) depict nanometric particles of IrO_2_. The minute size of the oxide’s grains indicate that the iridium dioxide itself obtained layer is characterized by quite a high surface area.

In the TEM scan of IrO_2_-NT material (Figure 1b), contrast black spots visible against the carbon nanotubes are the iridium dioxide nanoparticles. As can be seen, oxide particles effectively adhere to carbon nanomaterial, elevating the surface area of the material in comparison with standalone IrO_2_.

The TEM scan of IrO_2_-NT-POT (Figure 1c) revealed carbon nanotubes covered with single particles of iridium dioxide and poly(3-octylthiophene-2,5-diyl). It can be seen that agglomerates of iridium dioxide and poly(3-octylthiophene-2,5-diyl) cover the nanotubes of carbon material, creating a complicated structure of high surface area (Figure 1c). The designed triple composite material is characterized by a high surface area due to the complicated structure. The combination of three different substrates allowed the creation of one complex structure composite material and consequently to achieve the highest surface area of all designed solid-contact materials.

### 3.2. Potentiometric Response towards Hydrogen Ions

**Figure 2:**
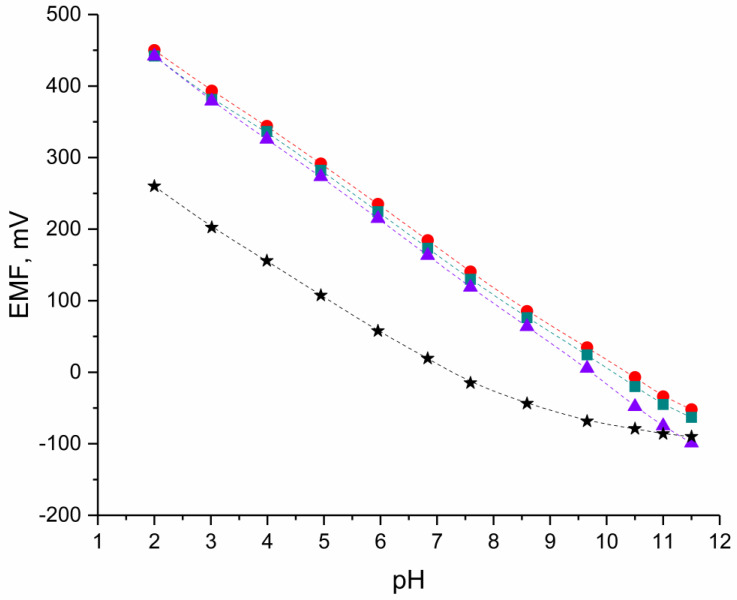
Exemplary potentiometric response ●-GC/hIrO_2_/H^+^-ISM, ■-GC/hIrO_2_+NTs/H^+^-ISM, ▲-GC/hIrO_2_+NTs+POT/H^+^-ISM and ★-GC/pH-ISM electrode after 24 h of electrodes’ conditioning in pH 3.

The ionic response of designed solid-contact hIrO_2_-electrodes was tested during potentiometric measurements in standard solutions of pH values from 2 to 12 in the presence of the group of control (coated-disc) electrodes. The electromotive force (EMF) was recorded during the time of three days of electrodes’ conditioning in pH 3 (after 24, 48, and 72 h). The exemplary potentiometric response is presented in the Figure 2 for one electrode of each group after 24 h of the electrodes’ conditioning. Average analytical parameters of designed electrodes calculated after 24, 48, and 72 h—the slope of the calibration curve, the standard potential, and the linear range, together with standard deviation values, are presented in the Table 1.

As expected, for solid-contact pH-selective electrodes the linear range—the range of pH in which the near-Nernstian response towards hydrogen ions is observed, was wider in contrast to coated-disc electrode. The slope of the calibration curve for designed solid-contact electrodes based on hydrous iridium dioxide was closer to the theoretical value (approx. 54 to 57 mV/pH) than for the electrode without the solid-contact layer (only 49 mV/pH). 

In addition, the repeatability of the electrodes’ response was better for electrodes with hIrO_2_-based materials since, for the GC/H^+^-ISM group, the standard deviation values calculated over three days were of higher values.

Within the groups of solid-contact electrodes with hydrous iridium dioxide (GC/hIrO_2_/H^+^-ISM) and double composite of hydrous iridium dioxide and carbon nanotubes (GC/hIrO_2_-NTs/H^+^-ISM), the potentiometric response towards hydrogen ions was comparable with similar parameters obtained in the same linear range—from pH 2 to 11. 

For the third group of solid-contact electrodes with triple composite material, the wider linear range was recognized (from pH 2 to 11.5). In addition, the smallest standard deviation values, describing the repeatability of electrodes’ response over three days of conditioning, can be attributed to the GC/hIrO_2_-NTs-POT/H^+^-ISM group. Implementing the triple composite material into the electrodes’ construction allowed us to obtain sensors of highly repeatable potentiometric response and the value of the slope of the calibration curve closest to the theoretical value in the pH range from 2 to 11.5.

Potential reversibility was tested in the set of standard solutions of pH values from 3 to 6. The measurement lasted 3 min in the solution of a certain pH, and the stability of potentiometric response was examined when changing the pH of the standard solution.

**Figure 3:**
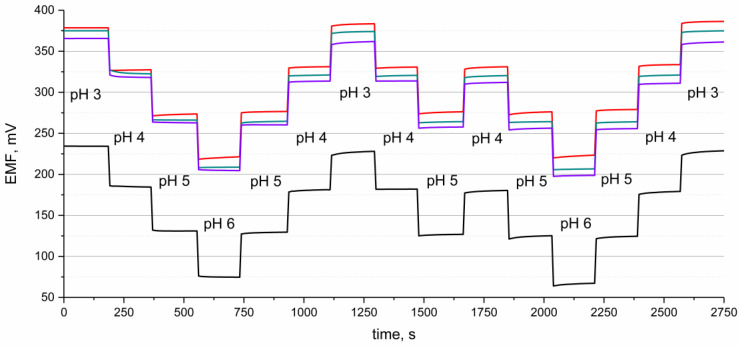
Potential reversibility of GC/hIrO_2_/H^+^-ISM (red line), GC/hIrO_2_+NTs/H^+^-ISM (green line), GC/hIrO_2_+NTs+POT/H^+^-ISM (purple line) and GC/H^+^-ISM (black line) electrode recorded in the buffer solution of pH 3, 4, 5 and 6, alternately.

As can be seen in the Figure 3, the potentiometric response is reversible for all tested groups of electrodes, and the EMF stabilizes immediately after the change in the pH value.

### 3.3. Potential Stability

**Figure 4:**
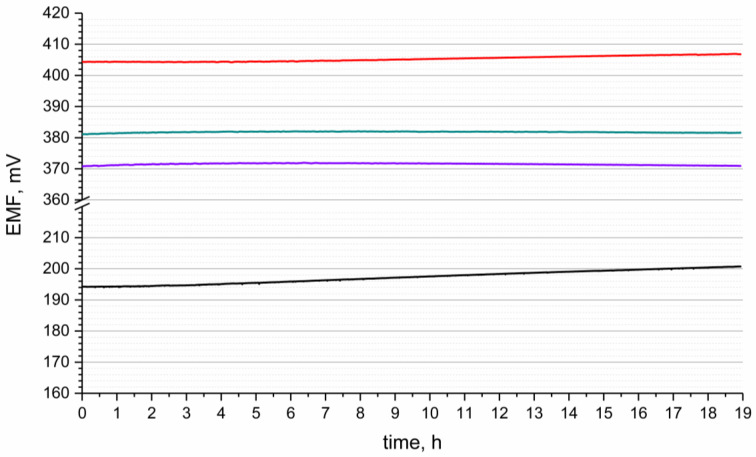
Potential stability of GC/hIrO_2_/H^+^-ISM (red line), GC/hIrO_2_+NTs/H^+^-ISM (green line), GC/hIrO_2_+NTs+POT/H^+^-ISM (purple line) and GC/H^+^-ISM (black line) electrode recorded in the buffer solution of pH 3.

The stability of potentiometric response of designed electrodes was tested for 19 hours in the standard buffer solution of pH 3. The course of the measurement with time is presented in the Figure 4. The measurement was conducted versus the single junction potential reference electrode—Ag/AgCl electrode with 3 M KCl solution. The potential stability was characterized by the potential drift parameter, which was calculated as ΔEMF/t ratio (ΔEMF—electromotive force change, t—time of measurement). The stability of potentiometric response was compared for each group of solid-contact hIrO_2_-based electrodes and the results were juxtaposed with the potential drift received for coated-disc electrode. 

For the solid-contact electrodes the results of potential drift values were as follows: 0.1 mV/h, 0.077 mV/h and 0.036 mV/h for GC/hIrO_2_/H^+^-ISM, GC/hIrO_2_-NTs/H^+^-ISM, and GC/hIrO_2_-NTs-POT/H^+^-ISM electrode, respectively. As noticed, the addition of carbon nanomaterial—and later on, conducting polymer—allows us to obtain electrodes of lower potential drifts (that is, higher potential stability). This phenomena was observed in our previous work on potassium-selective electrodes as for electrodes with standalone metal oxide (ruthenium dioxide) equaled to 0.085 mV/h [18] and 0.028 mV/h [19] and 0.077 mV/h [20] after implementing composite materials of this oxide with poly(3-octylthiophene-2,5-diyl) and Poly(3,4-ethylenedioxythiophene) Polystyrene Sulfonate, respectively.

In the literature for solid-contact pH-selective electrodes, the following values can be found: 2.4 mV/h [15] for poly(3,4-ethylenedioxythiophene)/poly(styrenesulfonate)—contacted electrode, 0.5 mV/h [17] for multiwalled carbon nanotubes—contacted electrode, and 0.15 mV/h [14] for hydrous ruthenium dioxide—contacted electrode. 

The significantly lower potential stability in contrast to the solid-contact electrodes, of 0.42 mV/h of coated-disc electrode GC/H^+^-ISM-ISM, is due to the blocked interface between the electronic and ionic conductor and the lack of the ion-to-electron transducer between the electrode and ion-selective membrane.

### 3.4. Redox Test

The EMF response for one electrode representing each group (three solid-contact hIrO_2_-based electrodes and one coated-disc electrode) was recorded in the set of solutions containing constant amount of a FeCl_2_ and FeCl_3_ redox couple (1 mM) with the logarithm of Fe^2+^/Fe^3+^ ratio equal to −1,−0.5, 0, 0.5, and 1. 

**Figure 5:**
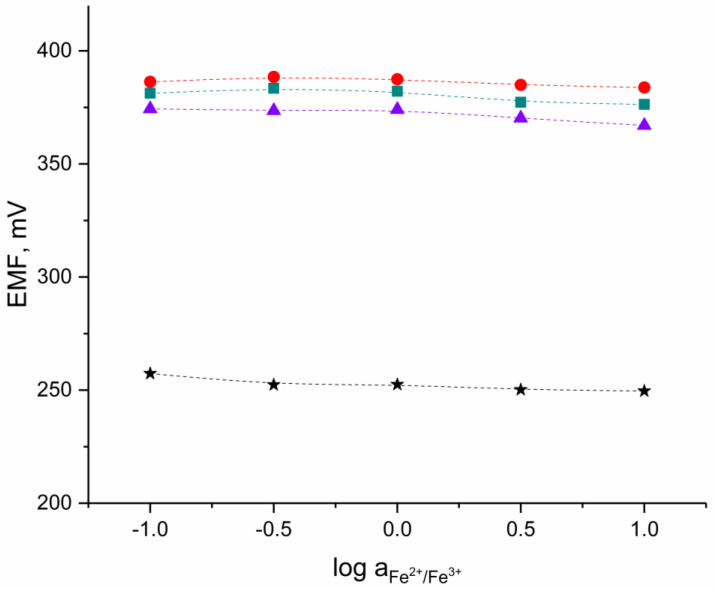
Redox test conducted in the Fe^2+^/Fe^3+^ redox couple solutions for ●-GC/hIrO_2_/H^+^-ISM, ■-GC/hIrO_2_-NTs/H^+^-ISM, ▲-GC/hIrO_2_-NTs-POT/H^+^-ISM and ★-GC/H^+^-ISM electrode.

As presented in the Figure 5, there was no redox response detected in all tested electrodes. The slight change of potential response was caused by various pH values in examined solutions rather than by a redox signal. 

Although the hydrous iridium dioxide-based materials are electronic conductors [21] and should exhibit clear redox response, the polymer-based ion-selective membrane is an electronic insulator and prevents the redox sensitivity of electrodes. Therefore, the examined sensors do not exhibit redox response. This also implicates the proper coverage of tested electrodes with polymeric membrane during the electrodes’ preparation with the drop-casting method.

### 3.5. Light Test

The sensitivity to varying light conditions determines the stability of the sensors during the measurement of different or changing light intensity. This test was performed because of the presence of POT in the solid-contact layer, as this polymer was characterized in the literature as light sensitive [3].

**Figure 6:**
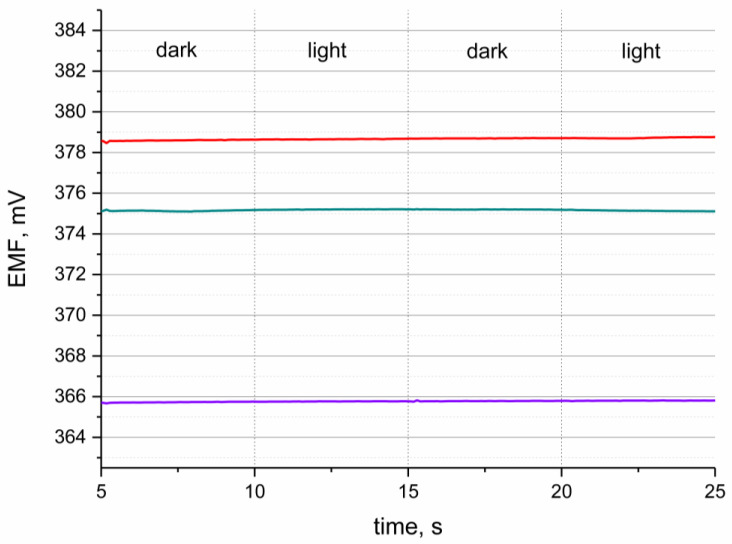
Light test conducted in the buffer solution of pH 2.5 for GC/hIrO_2_/H^+^-ISM (red line), GC/hIrO_2_+NTs/H^+^-ISM (green line), GC/hIrO_2_+NTs+POT/H^+^-ISM (purple line) and GC/H^+^-ISM (black line) electrode.

The light test was performed in the standard buffer solution of pH 3 during potentiometric measurement. The EMF was recorded while changing the intensity of light conditions from bright light to darkness. The procedure of the test is presented in the Figure 6. Sensors representing each group of designed electrodes were examined, and a stable potentiometric response was observed for all tested hIrO_2_-based electrodes. The potentiometric response was stable with time what depict that the light does not influence the performance of pH-selective sensors. 

The test proved that despite the presence of a light-sensitive conducting polymer, the designed sensors, with carbon nanotubes-poly(3-octylthiophene-2,5-diyl)-hydrous iridium dioxide triple composite material as a solid-contact, layer are characterized as light-insensitive. 

### 3.6. Water Layer Test

The water layer test was performed to evaluate the presence and the influence of the water thin film formed under the ion-selective membrane in the IrO_2_-based, pH-sensitive electrodes [22].

The experiment was performed according to the procedure proposed by Guzinski [16] in three steps. First, EMF was recorded in the primary ions (H^+^) solution of pH 2.5. Second, EMF was recorded in the secondary ions solution (0.1 M NaCl), and finally, it was recorded back in the standard H^+^ solution of pH 2.5. The same experiment was conducted for a coated-disc electrode. 

**Figure 7:**
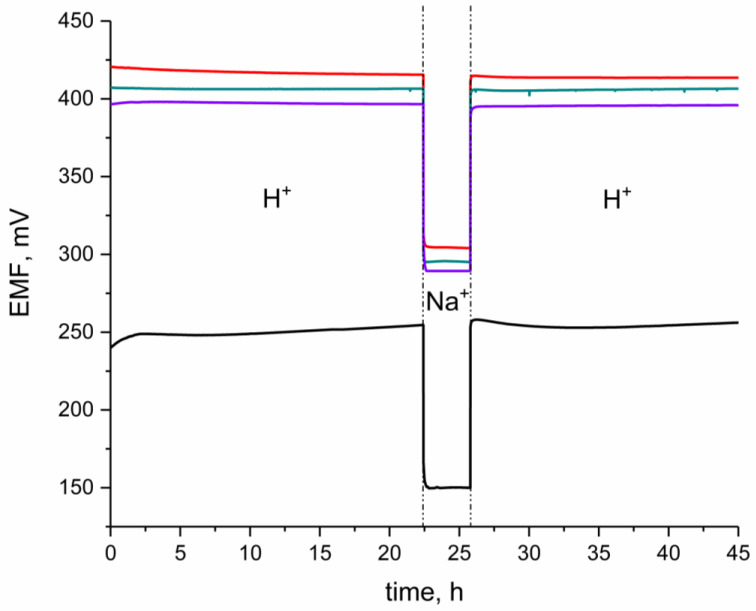
Water-layer test for GC/hIrO_2_/H^+^-ISM (red line), GC/hIrO_2_+NTs/H^+^-ISM (green line), GC/hIrO_2_+NTs+POT/H^+^-ISM (purple line) and GC/H^+^-ISM (black line) electrode. The test was conducted in the buffer solution of pH 3 (primary ion: H^+^) and 0.1 M NaCl (secondary ion: Na^+^).

As expected, in the EMF–time chart (presented in the Figure 6) of the GC/H^+^-ISM electrode, after the second step (contacting the interfering ion), the significant potential drift was observed. It took much longer for the coated-disc electrode to reach the equilibrium potential than it was observed for IrO_2_-based solid-contact electrodes. For all three groups of designed electrodes (GC/hIrO_2_/H^+^-ISM, GC/hIrO_2_-NTs/H^+^-ISM, GC/hIrO_2_-NTs-POT/H^+^-ISM) no potential drift was observed, and, even after contacting the 0.1 M NaCl solution, the potential response was stable through the time of measurement (as can be seen in the Figure 7), which proves the absence of the undesirable water layer.

**Figure 8:**
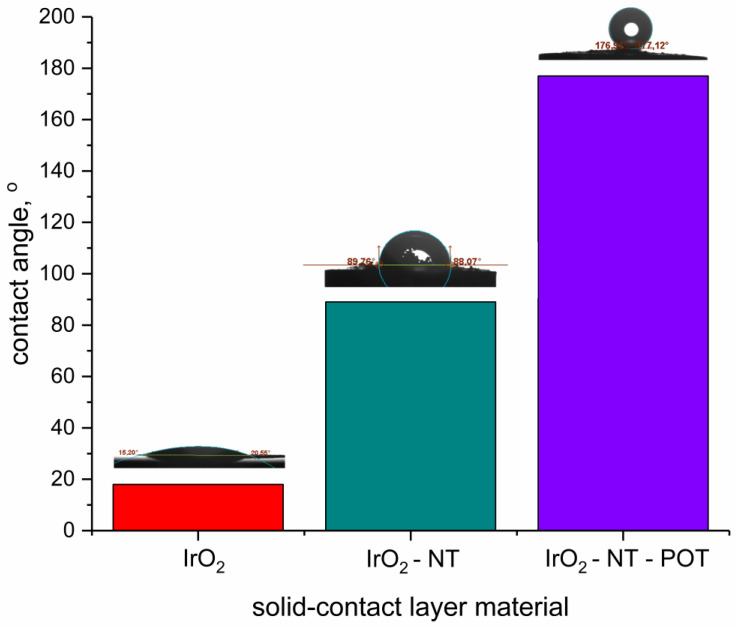
Contact angle values of materials for solid-contact layers for pH-selective electrodes (from left: standalone IrO_2_, double composite IrO_2_-NT and triple composite IrO_2_-NT-POT).

The main reason for the absenteeism of the water film under the pH-selective membrane in the solid-contact electrodes is the presence of the IrO_2_-based materials. The stability of the electrodes’ response during the water-layer test depends on the wetting properties of the solid-contact layer. The comparison of the contact angle values for all tested materials for solid-contact layers is presented in the Figure 8.

Standalone IrO_2_ is characterized by the low contact angle value (18 ± 1°), yet the addition of carbon nanotubes to the metal oxide allowed it to enhance the hydrophobicity of material by elevating the contact angle value (up to 89 ± 2°). The best performance during the experiment with primary and interfering ions (the best stabilization of potentiometric response) can be attributed to the GC/hIrO_2_-NTs-POT/H^+^-ISM group of electrodes characterized by the highest contact angle value (equal to 177 ± 3°). The addition of conducting polymer to the composite material changed the wetting properties of the material, making it highly hydrophobic, which, in consequence, contributed to the stability of the potentiometric response during the water layer test.

### 3.7. Electrical Parameters of PH-Sensors

Electrical capacitance, resistance, and potential drift in the forced current condition were calculated based on the results obtained using chronopotentiometry technique using the procedure proposed by Bobacka [12]. The results were presented in Table 2 for each group of pH-selective electrodes and compared. Chronopotentiograms were recorded while the current of 100 nA was forced to flow through the measurement cell. The measurement was conducted in six steps (three steps of recording potential response during +100 nA current flow and three steps of −100 nA current flow, alternately). The electrical parameters were calculated for each step, and average values together with standard deviation values are presented in the Table 2.

As presented in the Table 2, with the increasement of components in the solid-contact layer, the electrical capacitance increases and the resistance decreases. The best electrical parameters that are the highest electrical capacity and the lowest resistance and potential drift can be attributed to the group of electrodes with the triple composite layer.

## 4. Discussion

Analytical and electrical parameters of designed pH-selective electrodes based on hydrous iridium dioxide and its composites were compared with other electrodes of the same type presented so far in the literature. The compilation is presented in Table 3.

As presented in the table, electrodes with standalone IrO_2_, double composite IrO_2_-NT, and triple composite IrO_2_-NT-POT as the solid-contact layer are characterized by the linear range complementary to the previous solutions presented in the literature. Applying iridium dioxide-based triple composite material allowed it to receive the calibration plot with the slope value equal to 57.18, which is in agreement with the theoretical Nernstian value. What should be emphasized here is that the obtained IrO_2_-NT-POT-contacted electrode is characterized with the outstanding repeatability represented with the standard deviation values from the averaged values of the slope.

Although the capacitance values are not the highest of all the presented solutions, the potential drift values are considerably lower, which depicts the remarkable stability of the potentiometric response of the designed iridium dioxide-based electrodes. 

## 5. Conclusions

It was reportedly noticed that the increase in the number of components of a solid-contact layer is beneficial for the performance of pH-selective electrodes. Each component contributed differently to the final performance of the designed sensors. 

The addition of carbon nanotubes to iridium dioxide allowed us to increase the value of electrical capacitance and enhance the potential stability of sensor.

The addition of a conducting polymer to the composite material changed the wetting properties of the material, making it highly hydrophobic, which in consequence contributed to the stability of the potentiometric response during the water-layer test.

All designed groups of pH-selective hIrO_2_-based sensors exhibit near-Nernstian response in the pH range between 2 and 11. The response towards hydrogen ions turned out to be repeatable and reversible within this range of pH values, and neither redox nor light sensitivity were detected. No presence of a water layer was detected in the solid-contact electrodes with IrO_2_- based materials.

## Figures and Tables

**Table 1:** Calibration curves parameters of pH-selective electrodes after 24, 48 and 72 h of conditioning (n = 3).

Electrode	Linear Range [pH]	Slope S ± SD [mV/pH]	Standard Potential E^0^ [mV]
GC/hIrO_2_/H^+^-ISM	2–11	54.12 ± 0.16	557 ± 2
GC/hIrO_2_-NTs/H^+^-ISM	2–11	54.40 ± 0.19	548 ± 1
GC/hIrO_2_-NTs-POT/H^+^-ISM	2–11.5	57.18 ± 0.07	554 ± 1
GC/H^+^-ISM	2–7	48.93 ± 0.99	352 ± 8

**Table 2:** Electrical parameters calculated for the linear part of the recorded chronopotentiograms with standard deviation values (n = 6 steps).

Electrode	Potential Drift dE/dt ± SD [μV/s]	Resistance R ± SD [kΩ]	Capacitance C ± SD [μF]
GC/hIrO_2_/H^+^-ISM	1531 ± 19	807 ± 8	66 ± 8
GC/hIrO_2_-NTs/H^+^-ISM	253 ± 3	577 ± 4	174 ± 12
GC/hIrO_2_-NTs-POT/H^+^-ISM	309 ± 14	299 ± 3	387 ± 17

**Table 3:** Electrical and potentiometric parameters compared for a group of hydrous iridium dioxide-contacted electrodes with other pH-selective solid-contacted electrodes.

Solid Contact Material	pH Linear Range	Slope [mV/pH]	Potential Drift [mV/h]	Capacitance [μF]	Reference
Polypyrrole doped with hexacyanoferrate(II) (PPy-Fe(CN))	2–12	56.9 ± 4.3	0.005	-	[23]
Multi-walled carbon nanotube (MWCNT)	2.89–9.90	58.8 ± 0.4	0.5	30	[17]
Polydopamine-carbon nano-onion (CNO-PDA)	1.50–10.50	60.1 ± 0.3	-	-	[24]
Polyaniline (PANI)	2–9	52.7 ± 1.1	-	-	[25]
Derivative of poly(3,4-ethylenedioxythiophene) (PEDOT-C_14_)	3–11	57.7 ± 0.2	-	-	[16]
poly(3,4-ethylenedioxythiophene)−poly(styrenesulfonate) (PEDOT-PSS)	5–10.3	55.7± 0.5	2.4	-	[15]
Hydrous ruthenium dioxide (RuO_2_)	2–12	59.31 ± 0.15	0.15	1120	[14]
Hydrous iridium dioxide (IrO_2_)	2–11	54.12 ± 0.16	0.1	66	this work
Iridium dioxide-carbon nanotubes (IrO_2_-NT)	2–11	54.40 ± 0.19	0.077	174	this work
Iridium dioxide-carbon nanotubes- poly(3-octylthiophene-2,5-diyl) (IrO_2_-NT-POT)	2–11.5	57.18 ± 0.07	0.036	387	this work

## Data Availability

Not applicable.
